# 3D genital shape complexity in female marine mammals

**DOI:** 10.1002/ece3.7269

**Published:** 2021-02-15

**Authors:** Dara N. Orbach, Charlotte A. Brassey, James D. Gardiner, Patricia L. R. Brennan

**Affiliations:** ^1^ Department of Life Sciences Texas A&M University‐ Corpus Christi Corpus Christi TX USA; ^2^ Department of Biological Sciences Mount Holyoke College South Hadley MA USA; ^3^ School of Science and the Environment Manchester Metropolitan University Manchester UK; ^4^ Department of Musculoskeletal and Ageing Science Institute of Lifecourse and Medical Sciences University of Liverpool Liverpool UK

**Keywords:** alpha shape, genital, marine mammal, sexual selection, vaginal lumen

## Abstract

Comparisons of 3D shapes have recently been applied to diverse anatomical structures using landmarking techniques. However, discerning evolutionary patterns can be challenging for structures lacking homologous landmarks. We used alpha shape analyses to quantify vaginal shape complexity in 40 marine mammal specimens including cetaceans, pinnipeds, and sirenians. We explored phylogenetic signal and the potential roles of natural and sexual selection on vaginal shape evolution. Complexity scores were consistent with qualitative observations. Cetaceans had a broad range of alpha complexities, while pinnipeds were comparatively simple and sirenians were complex. Intraspecific variation was found. Three‐dimensional surface heat maps revealed that shape complexity was driven by invaginations and protrusions of the vaginal wall. Phylogenetic signal was weak and metrics of natural selection (relative neonate size) and sexual selection (relative testes size, sexual size dimorphism, and penis morphology) did not explain vaginal complexity patterns. Additional metrics, such as penile shape complexity, may yield interesting insights into marine mammal genital coevolution. We advocate for the use of alpha shapes to discern patterns of evolution that would otherwise not be possible in 3D anatomical structures lacking homologous landmarks.

## Graphical abstract

We describe unprecedented complexity of 3D genital shape in female marine mammals and explore potential evolutionary drivers. Cetaceans have the most complex vaginal shapes, followed by sirenians, then pinnipeds.

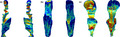

## 1 INTRODUCTION

Sexual selection, and natural selection to a lesser extent, can influence genital shape (Hosken & Stockley, 2004; Langerhans et al., 2005). The diversity, complexity, and rapid evolution of male genitalia has been well documented in many taxa (Eberhard, 1985; Hosken & Stockley, 2004). Female genital evolution, in contrast, has historically received limited scientific investigation, partly because female reproductive organs were thought to show low patterns of variation (Ah‐King et al., 2014). This oversight has hindered explorations of the intricate dynamics between the form and function of genitalia and thereby constrained some advancements in sexual and natural selection theory. Over the past decade, research on the morphological diversity of female genitalia has been revitalized using rigorous quantitative approaches that focus on overall shape rather than traditional linear measurements. Measures of shape tend to provide more information and demonstrate increased patterns of divergence compared with size metrics of genital traits (McPeek et al., 2008; Rohlf & Marcus, 1993; Slice, 2007). Among vertebrates, divergent female reproductive tract shapes have been found in snakes (Showalter et al., 2014), waterfowl (Brennan & Prum, 2012), sharks (Hedrick et al., 2019), and cetaceans (Orbach et al., 2018). Such shape analyses have used 2D geometric morphometric (GM) approaches, where homologous morphological landmarks were applied across photographs of specimens and subjected to Procrustes superimposition to remove the effects of translation, rotation, and scale. Comparison of 2D and 3D GM of female genitalia in spiny dogfish sharks (*Squalus acanthias*) found high congruence between data derived using the 2D and 3D approaches, although only 3D revealed significant patterns of asymmetry that may have biological relevance during pregnancy (Hedrick et al., 2019). While 2D GM can capture some aspects of shape, particularly in plate‐like structures, complex 3D structures require a different approach. With advances in bioimaging technologies, the application of 3D shape analyses to investigate relationships between form and function is rapidly growing (O'Higgins et al., 2011). Yet, landmark‐based 2D and 3D GM remain limited in their application to morphologically disparate structures with irregular shapes or lacking homologous landmarks, such as complex genital structures.

Geometric shape complexity analyses offer alternative metrics to GM for quantitative shape examination and can be thought of as the number of simple shape primitives required to adequately represent a given structure. Although morphological complexity can be difficult to assess and quantify, several continuous metrics exist (McLellan & Endler, 1998). Alpha shapes consist of a family of shapes fitted to a set of underlying points. 3D shape complexity was quantified without using landmarks in mammalian bacula, produced congruent results with other metrics of complexity (Gardiner et al., 2018), and revealed patterns of variation related to mating system (Brassey et al., 2020). Alpha shapes range from a very coarse convex‐hull fit to tightly‐fitting “shrink‐wraps.” The tightness of the fit is determined by a refinement coefficient, with small coefficient values reflecting tightly fitted shapes. Complex structures are defined as requiring a tight fit to match the original volume of the underlying mesh (Gardiner et al., 2018). Alpha shape analyses can be used to consistently and objectively quantify variation in shape complexity in irregular shapes lacking homologous landmarks (Gardiner et al., 2018) and can therefore be applied to assess the diversity and complexity of biological structures that are challenging to quantify but offer important insights into evolution.

The reproductive tract shapes of female cetaceans (whales, dolphins, and porpoises) represent a thus far unparalleled level of diversity in female genital morphology within a vertebrate clade (Orbach, Marshall, et al., 2017, 2018). Cetaceans possess vaginal folds, which are protrusions of the vaginal wall into the vaginal lumen, that vary in number, shape, size, and positioning across species (Orbach et al., 2018; Orbach, Marshall, et al., 2017). These vaginal folds are stiffer than other reproductive tract tissues (Orbach et al., 2019) and can physically occlude the penis during copulation, potentially providing females with a mechanism to control paternity (Orbach, Kelly, et al., 2017). Two‐dimensional GM indicated that vaginal and cervical shape diversity was influenced by ontogenetic and allometric factors in cetaceans, but not by neonate size or residual testes size (Orbach, Marshall, et al., 2017). However, qualitative assessments of the 3D vaginal lumen shape and penis shape of postmortem specimens in a few species of marine mammals have suggested close shape correspondence, coevolution, and varying complexity across species (Orbach et al., 2020; Orbach, Kelly, et al., 2017). Therefore, 3D shape analysis may elucidate the selection force(s) that act(s) on genital morphology and is not detectable in 2D. The complex shapes of cetacean vaginal lumens and lack of homologous landmarks in 3D models have hindered the use of landmark‐based methods to quantify variation across the clade. We use alpha shape analyses to quantify complexity in 3D vaginal shape across cetaceans and other marine mammals. Convergent evolution in body form and function is prevalent across marine mammals. Therefore, we also explored vaginal shape morphology in noncetacean marine mammals to assess the potential role of aquatic living as an evolutionary driver of shape complexity. As pinnipeds and sirenians also mate in the marine environment but do not have vaginal folds, we predict that non‐cetacean marine mammals will have less complex vaginal morphologies than cetaceans and that phylogenetic signal will be strong among marine mammals. We also predict that vaginal shape complexity will positively correlate with metrics of precopulatory sexual selection (sexual size dimorphism), copulatory sexual selection (penile morphology), and postcopulatory sexual selection (relative testes size), but not natural selection (relative neonate size at birth).

## 2 MATERIAL AND METHODS

### 2.1 Data collection

The intact reproductive tracts (from the ovaries to the external urogenital slit) of naturally deceased female marine mammals were collected opportunistically by marine mammal stranding networks and research institutions in the United States and New Zealand. Sexually immature (juvenile) and mature specimens were frozen immediately and transferred to necropsy facilities located at Mount Holyoke College. Information on the total body length of the animals and sexual maturity state (based on regional asymptotic body lengths or presence of *corpora albicantia* / *lutea* on the ovaries; (Perrin et al., 1984) was provided by the contributing institutions (Appendix S1).

Reproductive tracts were thawed and suspended with the uterine horns down and a ligature around the cervix to separate the vagina. Vaginal lumens were filled with Mold Star® 16 FAST or Elite HD^TM^ light body dental silicone to make endocasts. The silicone endocasts were carefully extracted to prevent artifacts or tears and to identify the ventral plane of orientation. Duplicate vaginal endocasts were made of some specimens, and as shapes were consistent, the original endocast was used to generate a 3D model using photogrammetry. A Canon EOS Rebel T5i camera with 100 mm lens and a set of four LED lights were used to take overlapping photographs of each endocast and capture the entire surface. Models were reconstructed in 3DF Zephyr lite (3Dflow SRL) and scaled.

### 2.2 Alpha shape analyses

The original application of alpha shapes to quantify 3D shape complexity used a volumetric computed tomography (CT) dataset, in which biological structures were represented by both external and internal data points (Gardiner et al., 2018). As photogrammetry meshes are composed solely of surface vertices, they were imported into MATLAB (Mathworks Inc.) and internally filled with a random distribution of points. Points were generated at random within the mesh's bounding box, and checked using “in_polyhedron” script of Jaroslaw Tuszynski (www.mathworks.com/matlabcentral/fileexchange/48041‐in_polyhedron) to confirm that each point was located inside the model's volume as defined by the surface mesh, until the point cloud contained a minimum of 250,000 points.

The analysis followed the methods described by Gardiner et al., (2018). Briefly, point clouds were down‐sampled to 100,000 points prior to shape fitting. A suite of alpha shapes was fitted to each specimen, ranging from an extremely coarse convex‐hull to a highly refined form as defined by the alpha radius (*α*). To account for variation in absolute size, *α* was scaled by a reference length specific to each model as:

$$\alpha =k\times l_{\text{ref}}$$

where *k* was the refinement coefficient and *l*_ref_ was the point cloud reference length. The same 200 values of *k* were chosen and evenly spaced between 0.1 to 10,000 on a logarithmic scale and all specimens (Appendix S2). Each specimen has a unique *l*_ref_, which was taken as the average distance of all points in the down‐sampled point cloud to their 100 nearest neighbors. After calculating a range of *α* values for each specimen, alpha shapes were fitted to the data using the “alphavol” function of Jonas Lundgren (www.mathworks.co.uk/matlabcentral/fileexchange/28851‐alpha‐shapes; Appendix S2). Each specimen was then described by a characteristic curve of alpha shape volume against refinement coefficient, with alpha shape volume decreasing as the fit became more refined (low values of *k;* Appendix S2). Such curves indicate the relative “scale” at which shape complexity is present within a given structure.

To explore how taxa differ in the scale of complexity present within the vaginal tract, we extracted the alpha shape volume (calculated as a percentage of “raw” photogrammetry mesh volume) for 6 values of the refinement coefficient (Appendix S2) across all specimens, representing 6 “scales” on which complexity may be measured. We chose 6 refinement coefficients equally spaced on the logarithmic scale to span the spectrum of highly refined alpha shapes to convex hulls. Sampled alpha shape volumes were then used as raw data for phylogenetically uncorrected principal components analyses (PCA) using the “prcomp” function of R (R Core Development Team, 2017). PCAs were conducted on both the “all‐individuals” dataset (to account for intraspecific variation) and on species means. Variables were scaled to have a zero mean and unit variance prior to analyses.

We calculated the optimal refinement coefficient as the value of *k* producing an alpha shape volume equal to the volume of the photogrammetry mesh (Appendix S2). Optimal *k* was identified by an optimization approach using the “fminsearch” function of MATLAB's optimization toolbox. We define “alpha complexity” as 1/optimal *k* (such that lower alpha complexity values reflect “simpler” shapes) and use this metric in all subsequent statistical analyses.

We also produce 3D heatmap meshes for the first time to further assist in the interpretation of alpha complexity scores. Each vertex of the mesh is assigned a value equal to the refinement coefficient of the coarsest alpha shape fit to which it contributes. Mesh faces are colored according to an average of their neighboring vertex values. Low complexity regions resolved by coarse alpha shapes are represented by cool colors while high complexity regions resolved only in tight alpha shape fits are represented by warm colors. These heatmaps are an advance from a single “optimal” complexity value for a whole structure and highlight specific anatomical regions of complexity that further facilitates evolutionarily meaningful morphological comparisons across taxa.

### 2.3 Phylogenetic considerations

To account for phylogenetic relationships within the dataset, statistical analyses were conducted within a phylogenetic comparative framework. A time‐calibrated phylogenetic tree was compiled from the literature with order‐level topology based on Foley et al., (2016), cetacean relationships derived from Zurano et al., (2019), and pinnipeds pruned from the carnivoran 10kTrees consensus tree (https://10ktrees.nunn‐lab.org/Carnivora/). The degree of phylogenetic signal present in the data was quantified as Pagel's lambda, estimated using the “phylosig” function of the R package “phytools” (Revell, 2012). Ancestral states were reconstructed using the “fastAnc” function of the same package.

### 2.4 Alternative selection pressures

We compiled the reported average lengths of neonates and their mothers at parturition from the literature (Appendix S3). These variables provide a proxy of a potential role of natural selection on vaginal shape complexity through parturition. Vaginal length at parturition is seldom reported in the literature. Phylogenetic generalized least squares (PGLS) regressions were conducted using the “gls” function of the “nlme” R package (Pinheiro et al., 2020), using a “corPagel” correlation structure (fixed = F) from the “ape” package (Paradis et al., 2004). A multiple regression was conducted with neonate length and mother body length as independent variables, thus providing a metric for residual neonate length.

The possible influences of sexual selection on vaginal shape complexity were tested by independently exploring residual testes size as a proxy for sperm competition risk, sexual size dimorphism, and a qualitative score of penile tip morphology. We compiled the largest reported testes mass (combined left and right) and maximum male body mass for all species in our study from published literature (Appendix S3); we used the largest relative testes mass per species to minimize error associated with seasonal variation in testes size. Phylogenetic generalized least squares (PGLS) regressions and multiple regression were conducted using the same approach as for neonate and mother body lengths, thus providing a metric of residual testes mass as a proxy for post‐copulatory sexual selection (Gage & Freckleton, 2003). Data on maximum male body lengths were compared with the body lengths of the females in our study to explore the possible role of sexual size dimorphism (male body length/female body length) driving vaginal complexity (Appendix S3). We reported maximum male body lengths from the literature and female mean body lengths from our samples to underscore the potential influence of sexual size dimorphism. All data were log_10_ transformed prior to analyses. We excluded *Mesoplodon densirostris* from the analysis because no testes mass or mother length at parturition data were available. To categorize the shape of the penis tip, we used data from our physical collection or from published photographs (Appendix S4). Penises were categorized as filiform (slender and filament‐shaped), tapered (distal tip flattened and gradually reduced in thickness), or blunt‐end (rounded tip with similar thickness to the shaft; Appendix S4). A phylogenetic ANOVA was conducted using the “phylANOVA” function of “phytools” to test for differences in mean alpha complexity based on penile morphological traits. The mean alpha shape volume per species was calculated at 6 values of refinement coefficient (as above) and subjected to a phylogenetic principal component analysis (pPCA) using the “phyl.PCA” function of phytools (mode=“cor”) using a lambda correlation structure. To test for statistical differences in alpha‐complexity morphospace occupation as a function of penis morphology, principal component scores were also input into a phylogenetically corrected MANOVA using the “aov.phylo” function of the package “geiger” (Pennell et al., 2014). All analyses were re‐tested, excluding the pinnipeds and sirenian, to explore the relationship between vaginal shape complexity and selective pressures within the phylogenetically constrained group of cetaceans.

## 3 RESULTS

A total of forty specimens was included in our study, represented by fourteen species of cetacean (*n* = 32 specimens), four species of pinniped (*n* = 7 specimens), and one species of sirenian (*n* = 1 specimen; Appendix S1).

**FIGURE 1:**
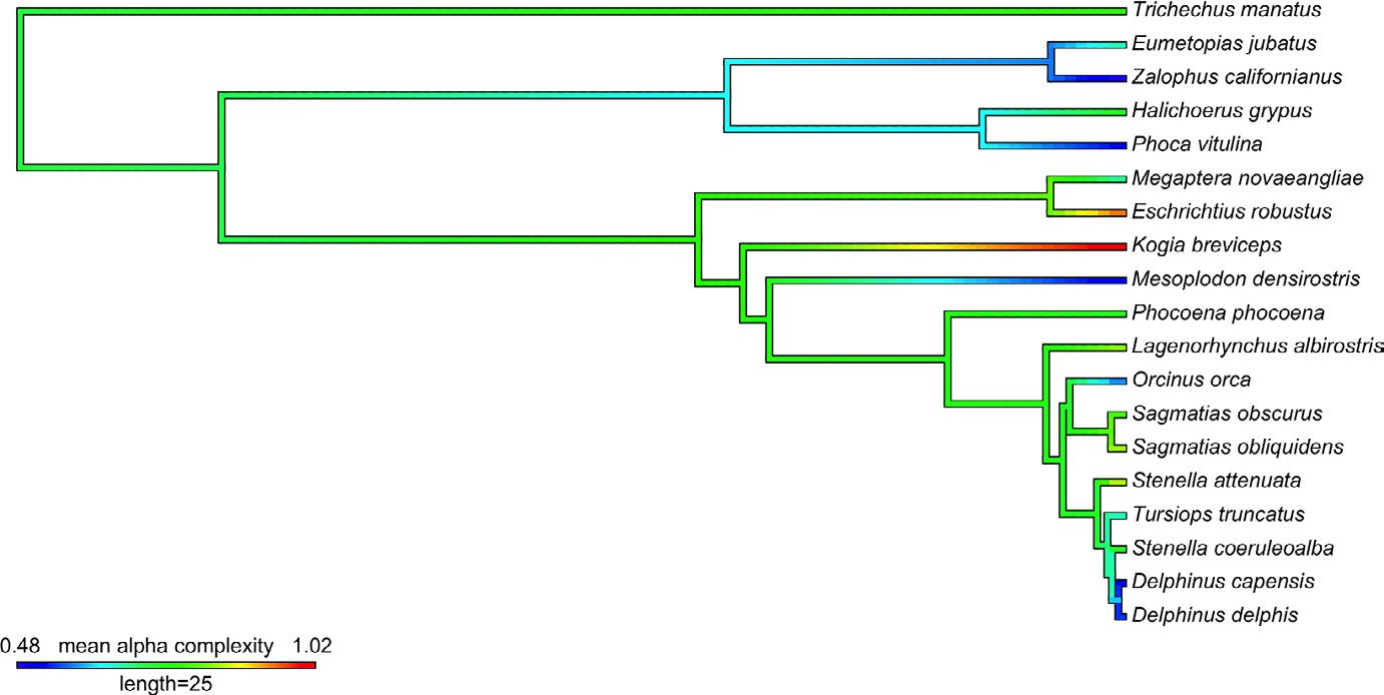
Ancestral state condition of 3D alpha complexity, reconstructed on a time‐calibrated composite phylogeny of marine mammals. Branch lengths in millions of years. Hot colors indicate higher vaginal shape complexity. Ancestral states were reconstructed using the “fastAnc” package of “phytools". Scale bar length represents 25 million years

Cetaceans had a range of endocast shapes and alpha complexities, including simple structures with only one indentation denoting a subtle vaginal fold, through to complicated shapes with spirals and many indentations of varying depths and sizes (Figure 1; Appendix S5). The pygmy sperm whale (*Kogia breviceps*) vagina was the most complex, with multiple deep protrusions of the vaginal wall. Although the genus *Lagenorhynchus* is no longer considered monophyletic (Vollmer et al., 2019), Pacific white‐sided dolphin (*Sagmatias obliquidens*), dusky dolphins (*Sagmatias obscurus*), and white‐beaked dolphins (*Lagenorhynchus albirostris*) were comparatively complex (Figure 1). The orca (*Orcinus orca)*, Blainville's beaked whale (*Mesoplodon densirostris*), and two species of common dolphin (*Delphinus capensis* and *D. delphis*), had comparatively simple vaginal lumens. Overall, pinnipeds had comparatively simple vaginal lumen shapes, with no spirals and few indentations. Otariids (fur seals and sea lions) were characterized by very low values of alpha complexity, as predicted (Figure 1; Appendix S5). Phocids (seals) also possess low 3D shape complexity despite some overlap with cetacean species. Contrary to predictions, the vaginal tract of the sirenian was found to be complex with an intermediate alpha complexity (Figure 1; Appendix S5). The manatee (*Trichechus manatus*) vaginal lumen was an intricate structure with extensive changes in diameter, a cup‐shape near the distal connection with the cervix, and a pronounced indentation midway through the vaginal canal made by a protruding structure akin to a vaginal fold. No significant phylogenetic signal in alpha complexity was detected across the phylogeny when calculated for all marine mammal taxa in our study, or within the cetacean subset (*λ* =< 0.001, *p* = 1).

**FIGURE 2:**
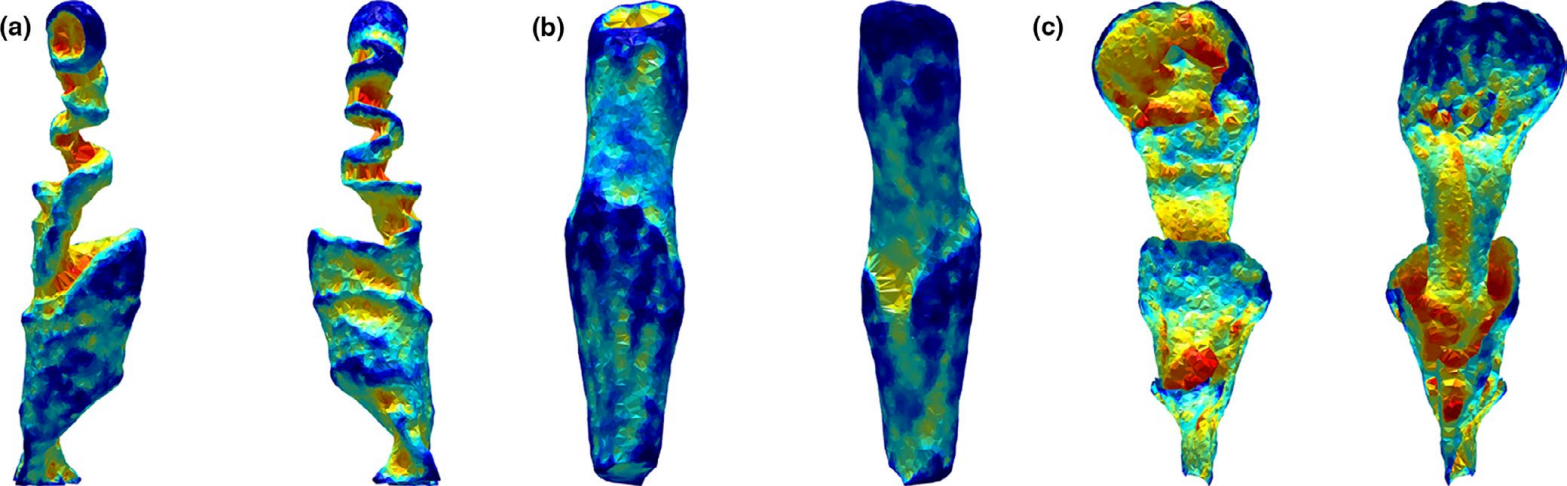
Three‐dimensional surface heatmaps of marine mammal vaginal endocasts of (a) Harbour porpoise (*Phocoena phocoena*), (b) California sealion (*Zalophus californianus*), and (c) Florida (*Trichechus manatus*). Endocasts are positioned cranial (cervix) up. The left image in each panel shows a ventral view while the right image shows a dorsal view. Cool colors represent anatomical regions that are resolved by comparatively coarse alpha fits, whereas areas contributing only to highly refined fits are represented by hot colors. Hot colors therefore illustrate the regions that are most influential in driving high alpha complexity. The resulting heatmaps are plotted onto the optimal refinement alpha shape fit for a given model

Three‐dimensional surface heatmaps indicate a heterogenous distribution of shape complexity across the endocasts, corresponding to discrete anatomical features within the vaginal lumen (Figure 2). High alpha complexities correspond to deep invaginations in the lumen (hot colors; protrusions of the vaginal wall or *os* cervix), whereas low alpha complexity correspond to non‐tapering regions with few invaginations (cold colors; Figure 2).

**FIGURE 3:**
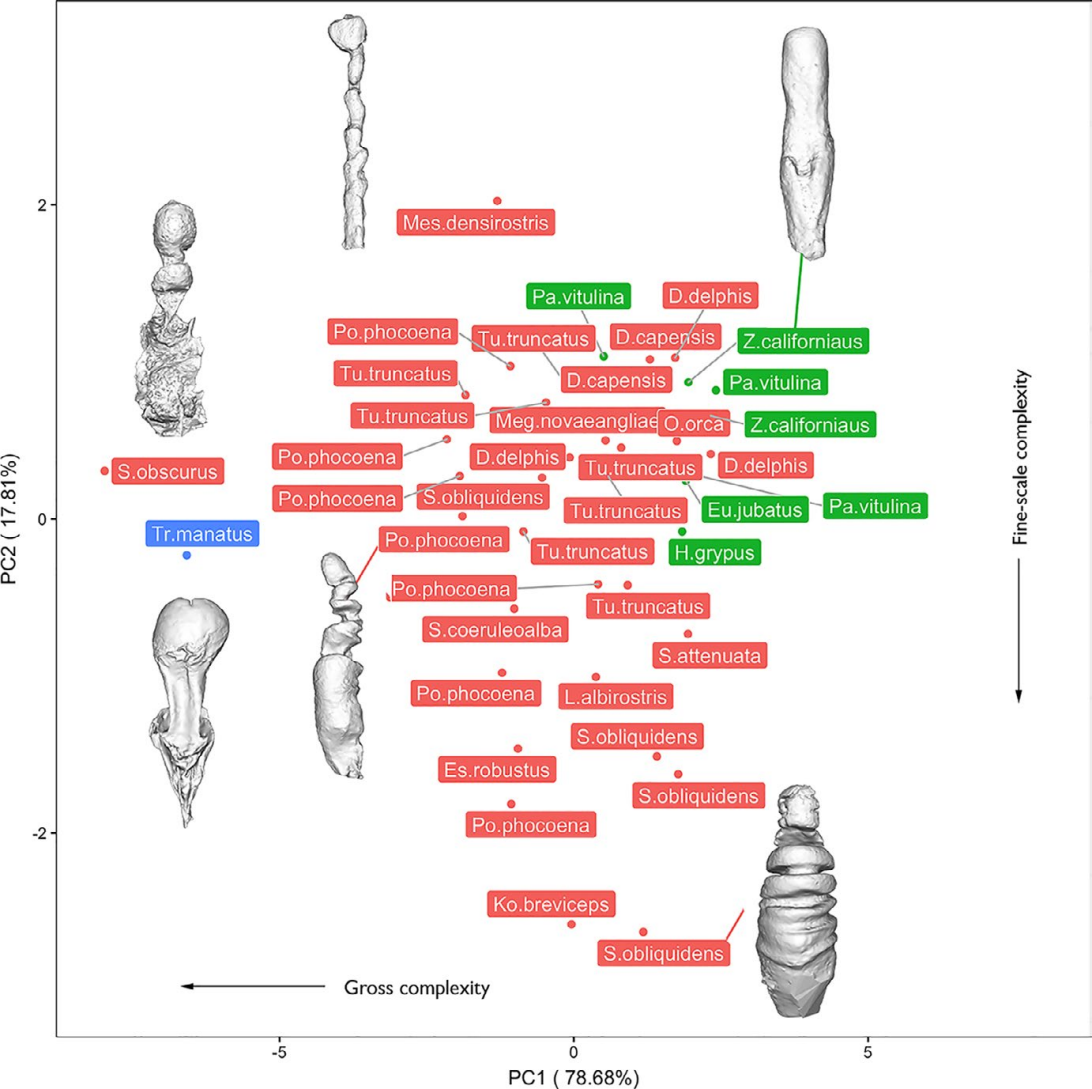
Uncorrected PCA conducted on raw alpha complexity dataset (including multiple individuals per taxa), with representative individuals displayed as 3D surface renderings. Pinnipeds are in green, cetaceans are in red, and the sirenian is in blue

Alpha shape volumes (calculated as a percentage of the original mesh volume) were extracted for 6 equally spaced refinement coefficients for individual specimens and subjected to a phylogenetically uncorrected principal component analysis. PC1 accounted for 79% of total variation and was negatively and heavily loaded with metrics of gross complexity extracted at coarse refinement coefficients (Figure 3). PC2 accounted for 18% of total variation and was negatively loaded with fine scale surface textural complexity (Figure 3). When repeated on species mean data, the resulting PCA was extremely similar in cumulative variations and variable loadings (Appendix S6). A phylogenetically corrected PCA conducted on a species means dataset was characterized by extremely low phylogenetic signal (*λ* =< 0.001) and therefore illustrated an identical distribution, with PC1 and PC2 negatively loaded with gross and fine‐scale complexity, respectively.

Cetaceans were widely distributed across the morphospace. Spanning PC1 (negatively associated with gross‐scale complexity), *Sagmatias obliquidens, Stenella attenuata,* and both species of *Delphinus* were characterized by high PC1 scores (low gross complexity), while *Sagmatias obscurus and Phocoena phocoena* were characterized by low PC1 scores (high gross complexity; Figure 3; Appendix S6). PC2 was negatively correlated with fine‐scale surface complexity, with *Mesoplodon densirostris* characterized by high PC2 scores (low surface complexity) and *Kogia breviceps* possessing low PC2 scores (high surface complexity).

Pinnipeds clustered in shape complexity morphospace, scoring high in both PC1 and PC2 (i.e., low macro‐ and fine‐scale complexity; Figure 3; Appendix S6). The sirenian was a discrete outlier at the negative extreme of PC1, possessing a high degree of gross complexity driven by the presence of a large invagination of the vaginal wall and tapering of the cranial vagina prior to widening at the *os cervix* (Figure 3; Appendix S6). Overall, when species were represented by multiple individuals, some degree of species‐level grouping in complexity morphospace is apparent, yet considerable intraspecific variation exists (Figure 3, Appendix S7).

Using PGLS multiple regressions, we found that alpha complexity does not correlate to relative neonate length (a proxy for the possible action of natural selection on parturition), in accordance with our prediction (Table 1; Appendix S8). Contrary to our prediction that sexual selection would explain shape complexity, we found that alpha complexity did not correlate with relative testes mass (a proxy for the strength of postcopulatory sexual selection) or sexual size dimorphism (a proxy for precopulatory sexual selection; Table 1; Appendix S8). When analyses were conducted on a subset of the data including only cetaceans, the above relationships remained non‐significant. We found no significant correlation between sexual size dimorphism and relative testes mass (*p* = .48).

**TABLE 1:** Results of regression models of alpha complexity in relation to various predictor variables using standard generalized least squares (GLS). All analyses were conducted on log10 transformed data

Trait (*α* shape complexity)	*N*	Predictor	Slope ± *SE*	*T*	*p*
All taxa	18	Neonate length	0.51 ± 0.68	0.74	.47
		Mother length	−0.33 ± 0.56	−0.58	.57
All taxa	18	Testes mass	0.03 ± 0.03	0.95	.36
		Body mass	−0.00 ± 0.21	−0.09	.93
All taxa	18	Sexual size dimorphism	−0.36 ± 0.27	−1.36	.19
Cetaceans only	12	Neonate length	0.67 ± 0.88	0.76	.47
		Mother length	−0.51 ± 0.74	−0.70	.51
Cetaceans only	12	Testes mass	−0.05 ± 0.07	−0.71	.49
		Body mass	0.03 ± 0.04	0.70	.51
Cetaceans only	12	Sexual size dimorphism	−0.38 ± 0.38	−0.98	.35

**FIGURE 4:**
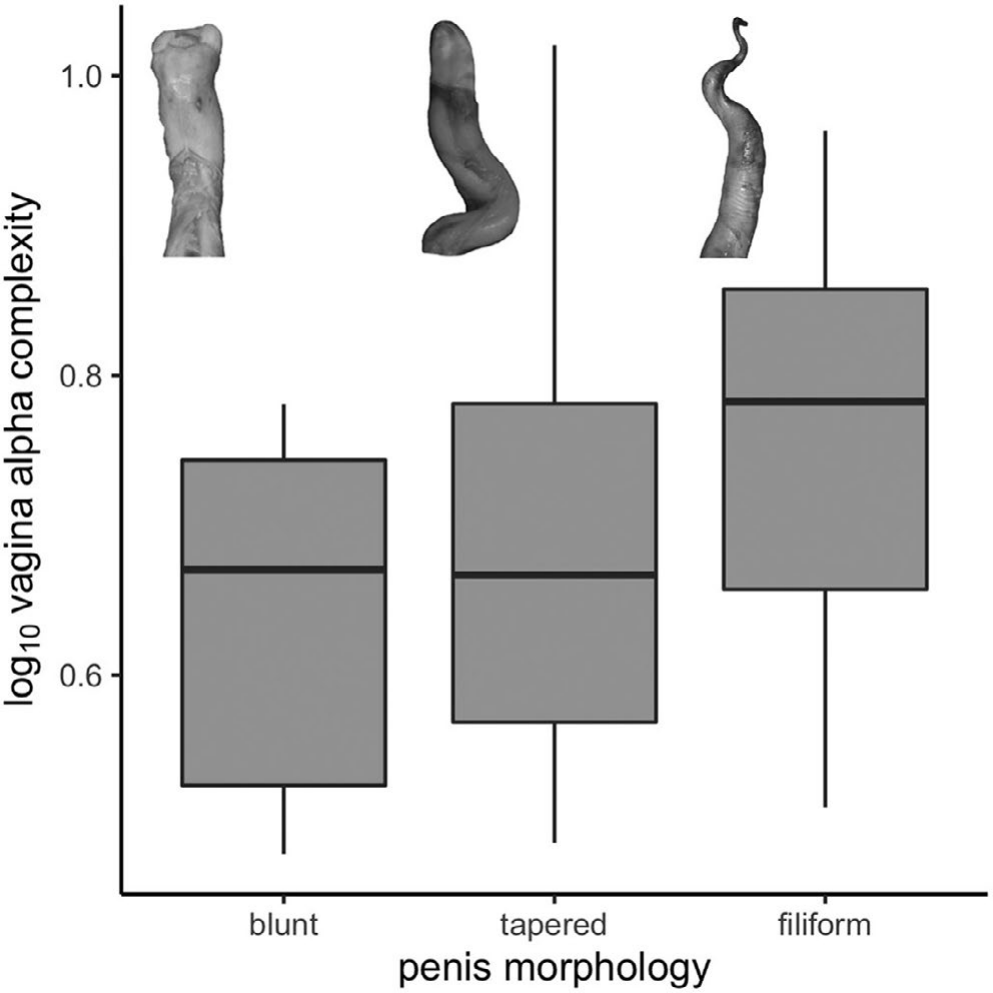
A boxplot illustrating the distribution of alpha complexity values of the vaginal tract in relation to the corresponding penile morphology

We also did not find a significant difference in mean alpha complexity of the vaginal tract among penile tip qualitative categories in our ANOVA (ANOVA: *F* = 0.67, *p* = .53; pANOVA: *F* = 0.67, *p* = .82; Figure 4). While species characterized by blunt‐ended and tapered penis tips typically appear to possess vaginal lumens with comparatively lower complexity than those with filiform penises, these differences are not statistically significant. Similarly, a MANOVA incorporating all PC scores did not find a significant difference in the occupancy of complexity morphospace by vaginal endocasts in relation to penile tip morphology (all specimens MANOVA: *F* = 0.67, *p* = .76; phyMANOVA: *F* = 0.67, *p* = .99; cetaceans MANOVA: *F* = 0.43, *p* = .84; phyMANOVA: *F* = 0.43, *p* = .94).

## 4 DISCUSSION

We describe and quantify 3D shape complexity in the female reproductive tract of mammals for the first time and assess patterns of variation within and across three marine clades. Quantitative alpha shape scores are consistent with qualitative patterns of shape complexity, which are reinforced by our novel application of 3D surface heatmaps. Marine mammals have a broad range of vaginal shape complexities that cannot easily be attributed to phylogeny, natural selection, or sexual selection, raising additional questions about the function of this diversity.

Vaginal lumen shape complexity is variable both within and among species of marine mammals. Cetaceans spanned the morphospace in both fine‐scale and gross complexity, although multiple representatives from a given species generally clustered. All 14 cetacean species had at least one vaginal fold that varied in number and size and contributed to alpha complexity, as demonstrated by the surface heatmaps. The phylogenetic signal of alpha complexity was weak, consistent with 2D vaginal shape analysis using a landmark‐based geometric morphometric approach (Orbach et al., 2018). As predicted, pinnipeds have simple vaginas with low vaginal complexity in terms of macro‐scale morphology and fine‐scale surface texture (Figure 3), regardless of whether they breed on the land (grey seals, California sealions, and Steller's sealions) or in the water (harbor seals). This suggests that mating environment may not influence vaginal shape complexity in pinnipeds. Given the diverse phylogenetic origins of cetaceans, pinnipeds, and sirenians, inclusion of their closest terrestrial relatives in future studies may advance our understanding of how mating environment influences the evolution of vaginal shape. The one sirenian specimen, the Florida manatee, lacks vaginal folds like pinnipeds, but has a surprisingly complex vaginal shape driven primarily by gross complexity. This complexity reflects the unusual morphology of the manatee vagina, with changes in diameter (Rodrigues et al., 2008), a protrusive hymen (Rodrigues et al., 2008), and a cup‐shaped distal tip that collectively correspond tightly with penis gross morphological shape (D. N. Orbach, P. L. R. Brennan, unpublished data). The 3D surface heatmaps indicate that invaginations in the manatee vagina are influential in driving high alpha shape complexity (Figure 2).

We report extensive intraspecific variation in alpha complexity of 3D vaginas that are not accounted for by phylogenetic relationships. Contrary to our prediction, 3D vaginal shape complexity did not correlate with our metrics of natural selection (relative neonate size at birth), precopulatory sexual selection (sexual size dimorphism), or postcopulatory sexual selection (relative testes size). These findings are similar to a previous study on 2D vaginal shape in cetaceans (Orbach et al., 2018). Perhaps alternative proxies, like oestrous state or neonate shape, may explain patterns of vaginal shape as hormone levels might alter biomechanical properties of tissues and as cetaceans are birthed tail‐first while pinnipeds are birthed head‐first. Future measurements of diverse proxies with larger sample sizes may yield new insights. While the filiform penis tips of marine mammals may be associated with complex vaginal shapes to facilitate navigation through the narrow vaginal lumen created by vaginal folds (Orbach et al., 2020; Orbach, Kelly, et al., 2017), we found no supporting evidence in the present study. However, our qualitative metric of penis tip shape may not adequately capture penis diversity. Future research that quantifies penis morphology may provide a better proxy to test the hypothesis that copulatory sexual selection drives vaginal shape diversity and support growing evidence of genital coevolution in marine mammals (Orbach et al., 2020; Orbach, Kelly, et al., 2017). As cetaceans are likely ubiquitously polygynandrous (Orbach & Würsig, 2019), mating system does not explain the extensive diversity observed in vaginal shape complexity within the clade. Similarly, the strength of polygynous mating systems, territory defence, or lek mating tactics does not appear to explain the patterns of pinniped vaginal complexity (Boness et al., 2006; Flatz et al., 2012; Parker & Maniscalco, 2014; Twiss et al., 1998).

The lack of support for our tests of variables to explain shape complexity may reflect our relatively small sample size. Caution is warranted as the addition or removal of a single specimen can alter our results (Appendix S8). While an increased sample size would be ideal, the opportunistic nature of collecting fresh, post‐mortem, sexually mature, female, marine mammal reproductive tracts imposes inherent limits (Orbach et al., 2016). Sample sizes are further curtailed as not all excised reproductive tracts are of suitable quality to generate an endocast and 3D model. Additionally, high intraspecific variation can increase the difficulty of detecting patterns.

Previous applications of alpha shape analyses to quantify genital shape complexity have typically relied exclusively on single metrics of “optimal complexity” (Gardiner et al., 2018), thereby overlooking potential spatial variation in complexity *within* a single structure and variation in complexity recorded at contrasting *scales*. Although our single metric of “optimal” alpha complexity does not correlate to proxies of natural or sexual selection, valuable details of vaginal shape complexity are elucidated by implementing new functionality into the alpha shape protocol. Additionally, the generation of 3D heatmap meshes now allows for an improved understanding of the spatial distribution of shape complexity and facilitates interpretation in the context of underlying anatomical features. Future iterations of alpha shapes and other complexity metric protocols will benefit from improving qualitative heatmaps to facilitate quantitative comparisons of regional complexity that allow for systematic subdivisions of a single structure into discrete anatomical regions for further analysis (Brassey et al., 2020). The extraction of alpha shape volumes at *multiple values of refinement coefficient* now allows for the generation of a PCA complexity “morphospace” and highlights the varying scales at which shape complexity may be present. Future research that analyzes whole alpha shape curves (of refinement coefficient against alpha shape volume) may be possible using statistical techniques such as “spm1d” (Pataky, 2010) and could provide novel insights into the complexity of a structure across *a range of scales*.

## CONFLICT OF INTEREST

None declared.

## AUTHOR CONTRIBUTIONS

**Dara Orbach:** Conceptualization (lead); Data curation (lead); Investigation (lead); Visualization (supporting); Writing – original draft (lead); Writing – review and editing (equal). **Charlotte A Brassey:** Formal analysis (lead); Methodology (lead); Software (lead); Validation (lead); Writing – original draft (lead); Writing – review and editing (equal). **James D. Gardiner:** Formal analysis (equal); Methodology (equal); Software (equal); Visualization (lead); Writing – review and editing (equal). **Patricia L. R. Brennan:** Conceptualization (lead); Data curation (supporting); Investigation (lead); Visualization (supporting); Writing – review and editing (equal).

## ETHICAL APPROVAL

Specimens in the United States of America were collected under National Marine Fisheries Service (NMFS) salvage permit letters to DNO. Specimens from New Zealand were imported to the United States of America under an institutional Convention on International Trade in Endangered Species of Wild Fauna and Flora permit (CITES; 14US690343/9).

## Supporting information

Supplementary Material

Supplementary Material

Supplementary Material

Supplementary Material

## Data Availability

Data archived in Texas A&M University‐ Corpus Christi's repository (https://tamucc‐ir.tdl.org/handle/1969.6/13).
