# Supplementary material for: 3D genital shape complexity in female marine mammals
Source: Ecol Evol. 2021 Feb 15;11(7):3210–8. doi: 10.1002/ece3.7269 (PMC8019040; doi:10.1002/ece3.7269)
Supplement: Supplementary file 1 — Supplementary Material [file ECE3-11-3210-s004.docx]

**Appendix 1.** Biological data on the specimens used in the study.

| **Specimen ID** | **Species** | **Age Class** | **Body Length (cm)** | **Stranding Location** | **Stranding Network** |
| --- | --- | --- | --- | --- | --- |
| 18Er03AprWI-01 | *Eschrictius robustus* | Subadult | 910 | Washington | Cascadia Research |
| C-364 | *Megaptera novaeangliae* | Subadult | 944.9 | California | The Marine Mammal Center |
| CRC 1573 | *Mesoplodon densirostris* | Adult | 431 | Washington | Cascadia Research |
| Calo 15-10 | *Kogia breviceps* | Adult | 286 | North Carolina | North Carolina State University CMAST |
| HMSC14-04-05-Sa | *Stenella attenuata* | Subadult | 199 | Oregon | Oregon State University |
| ScNEFL1722 | *Stenella coeruleoalba* | Adult | 200 | Florida | Florida Fish & Wildlife Conservation Commission |
| KXD0306 | *Delphinus capensis* | Adult | 201.8 | California | NOAA Southwest Fisheries Science Center |
| DSJ2385 | *Delphinus capensis* | Adult | 216 | California | NOAA Southwest Fisheries Science Center |
| VAQS20171042Dd | *Delphinus delphis* | Adult | 207.8 | Virginia | Virginia Aquarium Stranding Response |
| LMLDD2015OCT16 | *Delphinus delphis* | Adult | 182 | California | Long Marine Lab Stranding Network |
| HMSC18-04-19Dd | *Delphinus delphis* | Subadult | 176 | Oregon | Oregon State University |
| IFAW14-144Lalb | *Lagenorhynchus albirostris* | Adult | 242 | Massachusetts | International Federation of Animal Welfare |
| KS14-40Lo | *Sagmatias obscurus* | Adult | 175.5 | New Zealand | New Zealand Common Dolphin Project |
| HMSC15-08-18-Lo | *Sagmatias obliquidens* | Adult | 201.5 | Oregon | Oregon State University |
| RL160515.01 | *Sagmatias obliquidens* | Adult | 184.3 | California | NOAA Southwest Fisheries Science Center |
| RL160717.006 | *Sagmatias obliquidens* | Adult | 180 | California | NOAA Southwest Fisheries Science Center |
| RL160717.002 | *Sagmatias obliquidens* | Adult | 196.2 | California | NOAA Southwest Fisheries Science Center |
| SWC170242 | *Orcincus orca* | Adult | 548 | California | Seaworld |
| TtNEFL1517 | *Tursiops truncatus* | Adult | 263 | Florida | Florida Fish & Wildlife Conservation Commission |
| TtNEFL1801 | *Tursiops truncatus* | Subadult | 214 | Florida | Florida Fish & Wildlife Conservation Commission |
| VAQS20151079 | *Tursiops truncatus* | Subadult | 226.6 | Virginia | Virginia Aquarium Stranding Response |
| VAQS20151095 | *Tursiops truncatus* | Adult | 224.6 | Virginia | Virginia Aquarium Stranding Response |
| VAQS20161054 | *Tursiops truncatus* | Adult | 245.4 | Virginia | Virginia Aquarium Stranding Response |
| CALO 15-06 | *Tursiops truncatus* | Subadult | 194 | North Carolina | North Carolina State University CMAST |
| CALO18-01 | *Tursiops truncatus* | Adult | 264 | North Carolina | North Carolina State University CMAST |
| HMSC15-03-12-Pp | *Phocoena phocoena* | Subadult | 112 | Oregon | Oregon State University |
| WDFW2018-036 | *Phocoena phocoena* | Adult | 165 | Washington | Cascadia Research |
| HMSC16-08-27Pp | *Phocoena phocoena* | Adult | 160.5 | Oregon | Oregon State University |
| LMLPP2014SEPT17 | *Phocoena phocoena* | Adult | 171 | California | Long Marine Lab Stranding Network |
| IFAW15-035Pp | *Phocoena phocoena* | Subadult | 130 | Massachusetts | International Federation of Animal Welfare |
| SSW051617 | *Phocoena phocoena* | Adult | 166 | Washington | Cascadia Research |
| C434 | *Phocoena phocoena* | Adult | 174 | California | The Marine Mammal Center |
| NanHg040318 | *Halichoerus grypus* | Adult | 180 | Massachusetts | Nantucket Marine Mammal Conservation |
| HMSC18-04-22Ej | *Eumetopias jubatus* | Adult | 232 | Oregon | Oregon State University |
| HMSC18-03-11-Pv | *Phoca vitulina* | Adult | 143 | Oregon | Oregon State University |
| 2015-SJ042 | *Phoca vitulina* | Adult | 135 | Washington | The Whale Museum |
| 2015-SJ052 | *Phoca vitulina* | Adult | 150 | Washington | The Whale Museum |
| TMMC-CSL-13501 | *Zalophus californianus* | Adult | 165 | California | The Marine Mammal Center |
| TMMC-CSL-13502 | *Zalophus californianus* | Adult | 135 | California | The Marine Mammal Center |
| SWFTm1836b | *Trichecus manatus* | Adult | 326 | Florida | Florida Fish & Wildlife Conservation Commission |

**Appendix 2.** The process of calculating optimal refinement coefficient. A) Example set of alpha shapes fitted to three representative taxa. Fits increase in refinement from right and left, beginning with a convex hull. At highly refined fits, the volume of the alpha shape approaches that of the original surface mesh. B) Curves describing the decrease in alpha shape volume with decreasing refinement coefficient. Optimal fit (star symbol) is defined as the alpha shape possessing a volume equal to the original mesh (ratio = 1). Beyond this fit, alpha shapes begin to break down into an increasing number of separate elements until no fit can be achieved (sharp decline in volume ratios). Specimens possessing relatively high values of alpha shape volume extracted at low refinement coefficients (left side) are characterised by a ‘finer scale’ complexity (likely related to surface texture, pitting, etc.), while specimens at high (coarse) refinement coefficients (right side) are characterised by comparatively ‘coarse’ complexity (large ridges, grooves, invaginations, *etc.*). Open circles mark locations of sampling for PCA analysis. C) Magnified view of grey region in B, illustrating optimal refinement coefficient decreases and therefore “alpha complexity” (1/optimal *k*) increases from the comparatively simple *Z. californianus* to the complex *T. manatus.*

**Appendix 3.** Data on body lengths and weights used for regression analyses.

| **Species** | **Average Neonate Body Length at Parturition (cm)** | **Average Mother Body Length at Parturition (cm)** | **Maximum Combined Testes Mass (g)** | **Maximum Male Body Mass (g)** | **Male Maximum Body Length (cm)** | **Source** |
| --- | --- | --- | --- | --- | --- | --- |
| *Eschrictius robustus* | 455 | 1,345 | 67,500 | 40,823,000 | 1,460 | 1, 2, 3, 4 |
| *Megaptera novaeangliae* | 426 | 1,371 | 25,000 | 56,340,000 | 1,768 | 5, 6, 7 |
| *Mesoplodon densirostris* | 230 | Data unavailable | Data unavailable | Data unavailable | 580 | 4 |
| *Kogia breviceps* | 120 | 315 | 10,490 | 453,590 | 336 | 8, 9, 10 |
| *Stenella attenuata* | 105 | 201 | 2,896 | 119,000 | 257 | 11, 12, 13 |
| *Stenella coeruleoalba* | 96 | 221 | 450 | 156,000 | 256 | 14, 15 |
| *Delphinus capensis* | 89 | 204 | 6,414 | 127,000 | 255 | 16, 17 |
| *Delphinus delphis* | 72 | 183 | 7,940 | 163,000 | 260 | 4, 18, 19 |
| *Lagenorhynchus albirostris* | 115 | 245 | 2,810 | 345,200 | 315 | 4, 20, 21 |
| *Sagmatias obscurus* | 82 | 169 | 9,730 | 85,000 | 211 | 22, 23, 24, 25 |
| *Sagmatias obliquidens* | 94 | 180 | 1,118 | 198,000 | 205 | 4, 26, 27, 28, 29 |
| *Orcincus orca* | 233 | 548 | 46,200 | 10,488,000 | 980 | 4, 22, 30, 31 |
| *Tursiops truncatus* | 111 | 262 | 1,230 | 247,200 | 381 | 4, 22, 32 |
| *Phocoena phocoena* | 75 | 146 | 3,814 | 61,200 | 178 | 4, 33, 34 |
| *Halichoerus grypus* | Data unavailable | 169 | 120 | 204,000 | 265 | 35, 36, 37, 38 |
| *Eumetopias jubatus* | 96 | 226 | 177 | 938,000 | 330 | 35, 39, 40 |
| *Phoca vitulina* | 83 | 148 | 95 | 104,000 | 190 | 35, 41, 42, 43 |
| *Zalophus californianus* | 74 | 160^ | 113^ | 244,500 | 240 | 43, 44, 45 |
| *Trichecus manatus* | 125 | 315 | 2,350 | 706,000 | 253 | 46, 47, 48, 49 |

|  |
| --- |

Many references obtained from Dines et al. (2015) and Trites and Pauly (1998)

Dines, J. P., S. L. Mesnick, K. Ralls, L. May‐Collado, I. Agnarsson, and M. D. Dean. 2015. A trade‐off between precopulatory and postcopulatory trait investment in male cetaceans. Evolution 69(6):1560-1572.

Trites, A. W., and D. Pauly. 1998. Estimating mean body masses of marine mammals from maximum body lengths. Can. J. Zool*.* *76*(5):886-896.

1. Wolman A. A. 1985. Gray whale Eschrichtius robustus (Lilljeborg, 1861). Pp. 67-90 *in* S. H. Ridgway, and R. Harrison, eds. Handbook of marine ammals, Vol. 3. Academic Press, London, UK.
2. Robeck, T.R., K. J. Steinman, M. Yoshioka, E. Jensen, J. K. O’Brien, E. Katsumata, C. Gili, J. F. McBain, J Sweeney, and S. L. Monfort. 2005. Estrous cycle characterisation and artificial insemination using frozen–thawed spermatozoa in the bottlenose dolphin (*Tursiops truncatus*). Reproduction 129:659-674.
3. Rice D. W., A. A. Wolman, and H. W. Braham. 1984 The gray whale, *Eschrichtius robustus*. Mar. Fish. Rev. 46(4):7-14.
4. Klinowska, M. 1991. Dolphins, porpoises and whales of the world. *In* The IUCN Red Data Book. International Union for Conservation of Nature and Natural Resources, Gland, Switzerland, and Cambridge, U.K.
5. Chittleborough, R. G. 1958. Breeding cycle of the female humpback whale, *Megaptera nodosa* (Bonnaterre). Austral. J. Mar. Fresh. Res*.* 1-18.
6. Chittleborough, R. G. 1965. Dynamics of two populations of the humpback whale, *Megaptera novaeangliae* (Borowski). Mar. Fresh. Res*.* 16(1):33-128.
7. Clapham, P. J., S. Leatherwood, I., Szczepaniak, and R. L. Brownell Jr. 1997. Catches of humpback and other whales from shore stations at Moss Landing and Trinidad, California, 1919-1926. Mar. Mammal Sci. 13:368-394.
8. Ruiz, G. M. 1993. Male reproductive anatomy of the pygmy sperm whale, *Kogia breviceps*, and the dwarf sperm whale, *Kogia simus*, based on gross and histological bservations. MS thesis, University of South Florida, Tampa.
9. Ross, G. J. B. 1979. Records of pygmy and dwarf sperm whales, genus *Kogia*, from southern Africa, with biological notes and some comparisons. Annal. Cape Prov. Mus. Nat. Hist. 11(14):259-327.
10. Berta, A. 2015. Whales, dolphins, and porpoises: A natural history and species guide. University of Chicago Press, Chicago, IL.
11. Perrin, W. F., and A. A. Hohn. 1994. Pantropical spotted dolphin *Stenella attenuata*. Pp. 71-98 *in* S. H. Ridgway, and R. Harrison, eds. Handbook of marine mammals, Vol. 5. Academic Press, London, UK.
12. Hohn A., S. J. Chivers, and J. Barlow. 1985. Reproductive maturity and seasonality of male spotted dolphins, *Stenella attenuata*, in the eastern tropical Pacific. Mar. Mamm. Sci. 1(4):273-293.
13. Nishiwako, M., M. Nakajima, and T. Kamiya. 1965. A rare species of dolphin (*Stenella attenuata*) from Arari, Japan. Sci. Rep. Whales Res. Inst. 19:53-64.
14. Aguilar, A. 1991. Calving and early mortality in the western Mediterranean striped dolphin, *Stenella coeruleoalba.* Can. J. Zool. 69(5):1408-1412.
15. Perrin, W. F., C. E. Wilson, and F. I. Archer. 1994. Striped dolphin *Stenella coeruleoalba* (Meyen, 1833). Pp. 129-159 *in* S. H. Ridgway, and R. Harrison, eds. Handbook of marine mammals, vol. 5. Academic Press, London, UK.
16. Perrin, W. F. 2009. Common dolphins, *Delphinus delphis* and *Delphinus capensis*. Pp. 255-259 in W. F. Perrin, B. Wursig, and J. G. M. Thewissen, eds. Encyclopedia of marine mammals. Academic Press, Amsterdam, The Netherlands
17. Ngqulana, S. G., G. G. Hofmeyr, and S. Plön. 2017. Sexual dimorphism in long-beaked common dolphins (*Delphinus capensis*) from KwaZulu-Natal, South Africa. *J. Mamm.* *98*(5):1389-1399.
18. Chivers, S. J., W. L. Perryman, M. S. Lynn, T. Gerrodette, F. I. Archer, and K. Danil. 2016. Comparison of reproductive parameters for populations of eastern North Pacific common dolphins: *Delphinus capensis* and *D. delphis*. Mar. Mamm. Sci. 32(1):57–85.
19. Ross, G .J. B. 1979. The smaller cetaceans of the south east coast of Southern Africa. Annal. Cape Prov. Mus. Nat. Hist. 15(2):173-410.
20. Galatius, A., O. E. Jansen, and C. C. Kinze. 2013. Parameters of growth and reproduction of white-beaked dolphins (*Lagenorhynchus albirostris*) from the North Sea. Mar. Mamm. Sci. 29(2):348–355.
21. Sigurjónsson, J., and G. A. Víkingsson. 1997. Seasonal abundance of and estimated food consumption by cetaceans in Icelandic and adjacent waters. J. Northwest Atl. Fish. Sci. 22*:*271-287.
22. Perrin, W. F., and S. B. Reilly. 1984. Reproductive parameters of dolphins and small whales of the family Delphinidae. Rep. Int. Whal. Comm.*,* Spec. Issue 6:97-133.
23. Van Waerebeek, K., and A. J. Read. 1994. Reproduction of dusky dolphins, *Lagenorhynchus obscurus,* from coastal Peru. J. Mamm*.* 75(4):1054-1062.
24. Brownell Jr., R. L., and F. Cipriano. 1999. Dusky dolphin *Lagenorhynchus obscurus* (Gray, 1828). Pp. 85-104 *in* S. H. Ridgway, and R. Harrison, eds. Handbook of marine mammals, Vol. 6. Academic Press, San Diego, CA.
25. Dans, S. L., E. A. Crespo, S. N. Pedraza, and M. K. Alonso. 1997. Notes on the reproductive biology of female dusky dolphins (*Lagenorhynchus obscurus*) off the Patagonian coast. Mar. Mamm. Sci. 13(2):303-307.
26. Walker, W. A., S. Leatherwood, K. R. Goodrich, W. F. Perrin, and R. K. Stroud. 1986. Geographical variation and biology of the Pacific white-sided dolphin, *Lagenorhynchus obliquidens*, in the north-eastern Pacific. Pp. 441-465 *in* M. M. Bryden, and R. Harrison, eds. Research on dolphins*.* Clarendon Press, Oxford, UK.
27. Harrison, R. J., R. L. Brownell Jr., and R. C. Boice. 1972. Reproduction and gonadal appearance in some odontocetes. Pp. 361-429 in R.vJ. Harrison, ed. Functional anatomy of marine mammals, Vol. 1. Academic Press, London, UK.
28. Brownell Jr., R. L., W. A. Walker, and K. A. Forney. 1999. Pacific white-sided dolphin, *Lagenorhynchus obliquidens Gill*, 1865. Pp. 57-84 *in* S.H. Ridgway, and R. Harrison, eds. Handbook of marine mammals, Vol. 6. Academic Press, San Diego, CA.
29. Black, N. A. 2009.Pacific white-sided dolphin L*agenorhynchus obliquidens*. Pp. 817-819 *in* W. F. Perrin, B. Wursig, and J. G. M. Thewissen, eds. Encyclopedia of marine mammals. Academic Press, Amsterdam, The Netherlands
30. Mikhalev, Y. A., M. V. Ivashin, V. P. Savusin, and F. E. Zelenaya. 1981. The distribution and biology of killer whales in the Southern Hemisphere. Rep. Internat. Whal. Comm*.* 31:551-566.
31. Clark, S. T., D. K. Odell, and C. T. Lacinak. 2000. Aspects of growth in captive killer whales (*Orcinus orca*). Mar. Mamm. Sci. 16(1):110-123.
32. Storelli, M. M., and G. O. Marcotrigiano. 2000. Environmental contamination in bottlenose dolphin (*Tursiops truncatus*): Relationship between levels of metals, methylmercury, and organochlorine compounds in an adult female, her neonate, and a calf. Bull. Envir. Contam. Toxic. 64(3):333-340.
33. Learmonth, J. A., S. Murphy, P. I. Luque, R. J. Reid, I. A. P. Patterson, A. Brownlow, H. M. Ross, J. P. Barley, M. Begoña Santos, and G. J. Pierce. 2014. Life history of harbor porpoises (*Phocoena phocoena*) in Scottish (UK) waters. Mar. Mamm. Sci. 30:1427–1455.
34. Ólafsdóttir, D., G. A. Víkingsson, S. D. Halldórsson, and J Sigurjónsson. 2002. Growth and reproduction in harbour porpoises (*Phocoena phocoena*) in Icelandic waters. NAMMCO Sci. Pub. 5:195-210.
35. Scheffer, V. B., and K. W. Kenyon. 1963. Baculum size in pinnipeds. Z. Siiugetierk. 28:38-41.
36. Hammill, M. O., and J. F. Gosselin. 1995. Grey seal *(Halichoerus grypus*) from the Northwest Atlantic: Female reproductive rates, age at first birth, and age of maturity in males. Can. J. Fish. Aqua. Sci. 52:2757-2761.
37. Pomeroy, P. P., M. A. Fedak, P. Rothery, and S. Anderson. 1999. Consequences of maternal size for reproductive expenditure and pupping success of grey seals at North Rona, Scotland. J. Anim. Ecol. *68*(2):35-253.
38. Mansfield, A. W. 1977. Growth and longevity of the grey seal *Halichoerus grypus* in eastern Canada. International Council for the Exploration of the Sea, Marine Mammals Committee, C.M. 1977/ N:6.
39. Winship, A. J., A. W. Trites, and D. G. Calkins. 2001. Growth in body size of the steller sea lion (*Eumetopias jubatus*). J. Mamm. 82:500-519.
40. Calkins, D., E. F. Becker, and K. W. Pitcher. 1998. Reduced body size of female Steller sea lions from a declining population in the Gulf of Alaska. Mar. Mamm. Sci. 14:232–244.
41. Bigg, M. A. 1962. Age determination, reproduction, growth and population analysis of the Harbour seal, *Phoca vitulina richardi* gray. MSc Thesis, Department of Zoology, University of British Columbia.
42. Markussen, N. H., A. Bjørge, and N. A. Øritsland. 1989. Growth in harbour seals (*Phoca vitulina*) on the Norwegian coast. J*. Zool.* *219*(3):433-440.
43. McLaren, I. A. 1993. Growth in pinnipeds. Biol. Rev. Camb. Philos. Soc. 68:1–79.
44. Ferguson, S. H., and J. W. Higdon. 2006. How seals divide up the world: Environment, life history, and conservation. Oecologia 150: 318-329.
45. Gilmartin, W. G., L. R. Delong, A. W. Smith, J. C. Sweeney, R. W. de Lappe, R. W. Risebrough, L. A. Griner, M. D. Dailey, and D. B, Peakall. 1976. Premature parturition in the California sea lion. J. Wild. Dis. 12:104-1 15.
46. Borges, J. C. G., A. C. da Bôaviagem Freire, F. L. N. Attademo, I. de Lima Serrano, D. G. Anzolin, P. S. M. de Carvalho, and J. E. Vergara-Parente. 2012. Growth pattern differences of captive born Antillean manatee (*Trichechus manatus*) calves and those rescued in the Brazilian northeastern coast. J. Zoo Wild. Med*.* 43(3):494-500.
47. Moore, J. C. 1957. Newborn young of a captive manatee.*J. Mamm. 38*(1):137-138.
48. Reynolds III, J. E., S. A. Rommel, and M. E. Pitchford. 2004. The likelihood of sperm competition in manatees- explaining an apparent paradox. Mar. Mamm. Sci. 20(3):464-476.
49. Harshaw, L. T., I. V. Larkin, R. K. Bonde, C. J. Deutsch, and R. C. Hill. 2016. Morphometric body condition indices of wild Florida manatees (*Trichechus manatus latirostris*). Aqua. Mamm. 42(4):428.

**Appendix 4**. Penis tip shape of the species included in the study.

| **Species** | **Penis Tip Shape** | **Age Class** | **Source** |
| --- | --- | --- | --- |
| *Eschrichtius robustus* | Filiform | Adult | Unpublished data |
| *Megaptera novaeangliae* | Tapered | Adult | 1 |
| *Mesoplodon densirostris* | Filiform | Adult | 2 |
| *Kogia breviceps* | Tapered | Juvenille | Unpublished data |
| *Stenella attenuata* | Data unavailable | | |
| *Stenella coeruleoalba* | Tapered | Adult | 3 |
| *Delphinus capensis* | Tapered | Juvenille | 4 |
| *Delphinus delphis* | Tapered | Adult | Unpublished data |
| *Lagenorhynchus albirostris* | Filiform | Adult | Unpublished data |
| *Orcincus orca* | Filiform | Adult | Unpublished data |
| *Tursiops truncates* | Tapered | Adult | Unpublished data |
| *Phocoena phocoena* | Filiform | Adult | Unpublished data |
| *Sagmatias obscurus* | Filiform | Adult | Unpublished data |
| *Sagmatias obliquidens* | Filiform | Adult | Unpublished data |
| *Halichoerus grypus* | Blunt end | Adult | Unpublished data |
| *Eumetopias jubatus* | Blunt end | Adult | 5 |
| *Phoca vitulina* | Blunt end | Adult | Unpublished data |
| *Zalophus californianus* | Blunt end | Adult | Unpublished data |
| *Trichecus manatus* | Blunt end | Adult | Unpublished data |

|  |
| --- |

**Appendix 5.** Data arranged in descending order of alpha complexity. High values of alpha complexity indicate a comparatively ‘complex’ vaginal tract morphology.

| **Species** | **Individual ID** | **Alpha Complexity** | **Taxa** |
| --- | --- | --- | --- |
| *Kogia breviceps* | CALO1510 | 1.021 | Cetacean |
| *Sagmatias obliquidens* | RL16071 | 0.991 | Cetacean |
| *Eschrictius robustus* | 18Er03AprW1-01 | 0.964 | Cetacean |
| *Phocoena phocoena* | SSW051617 | 0.93 | Sirenian |
| *Sagmatias obliquidens* | RL160717 | 0.903 | Cetacean |
| *Stenella attenuata* | HMSC14-04-05 | 0.888 | Cetacean |
| *Sagmatias obliquidens* | RL160515 | 0.887 | Cetacean |
| *Lagenorhynchus albirostris* | IFAW14-144 | 0.843 | Cetacean |
| *Phocoena phocoena* | C-434 | 0.826 | Cetacean |
| *Stenella coeruleoalba* | ScNEFL1722 | 0.817 | Cetacean |
| *Phocoena phocoena* | IFAW15-035 | 0.79 | Cetacean |
| *Tursiops truncatus* | TtNEFL1517 | 0.784 | Cetacean |
| *Sagmatias obscurus* | KS1440 | 0.782 | Cetacean |
| *Trichecus manatus* | SWFTm1836b | 0.78 | Sirenian |
| *Tursiops truncatus* | CALO1801 | 0.763 | Cetacean |
| *Sagmatias obliquidens* | HMSC15-08-18 | 0.746 | Cetacean |
| *Halichoerus grypus* | NANHg040318 | 0.743 | Pinniped |
| *Phocoena phocoena* | HMSC16-08-27 | 0.734 | Cetacean |
| *Phocoena phocoena* | LMLPp2014Sept17 | 0.73 | Cetacean |
| *Delphinus delphis* | HMSC18-04-19 | 0.688 | Cetacean |
| *Phocoena phocoena* | WDFW2018-036 | 0.684 | Cetacean |
| *Tursiops truncatus* | VAQS20161054 | 0.682 | Cetacean |
| *Megaptera novaeangliae* | C-364 | 0.674 | Cetacean |
| *Phoca vitulina* | HMSC18-03-11 | 0.671 | Pinniped |
| *Eumetopias jubatus* | HMSC18-04-22 | 0.67 | Pinniped |
| *Tursiops truncatus* | VAQS20151079 | 0.631 | Cetacean |
| *Tursiops truncatus* | TtNEFL1801 | 0.625 | Cetacean |
| *Tursiops truncatus* | VAQS20151095 | 0.61 | Cetacean |
| *Phocoena phocoena* | HMSC15-03-12 | 0.56 | Cetacean |
| *Tursiops truncatus* | CALO1506 | 0.576 | Cetacean |
| *Orcinus orca* | SWC170242 | 0.571 | Cetacean |
| *Delphinus delphis* | LMLDd2015Oct16 | 0.563 | Cetacean |
| *Phoca vitulina* | 2015SJ052 | 0.533 | Pinniped |
| *Mesoplodon densirostris* | CRC1573 | 0.511 | Cetacean |
| *Zalophus californianus* | TMMC-CSL13502 | 0.494 | Pinniped |
| *Delphinus capensis* | KXD0306 | 0.488 | Cetacean |
| *Delphinus capensis* | DSJ2385 | 0.488 | Cetacean |
| *Zalophus californianus* | TMMC-CSL13501 | 0.468 | Pinniped |
| *Phoca vitulina* | 2015-SJ042 | 0.428 | Pinniped |
| *Delphinus delphis* | VAQS20171042 | 0.427 | Cetacean |

**Appendix 6.** Uncorrected PCA conducted on species mean dataset. Pinnipeds are in green, cetaceans are in red, and the sirenian is in blue.

**Appendix 7.** High intraspecific variation in the vaginal tract of harbour porpoises (*Phocoena phocoena*)*.* The figure shows the dorsal aspect of seven harbour porpoise vaginal lumens, all oriented with the vaginal opening at the bottom. Calculated alpha shape complexity increases from left to right (α: 0.560-0.930).

**Appendix 8**. Results of regression models of alpha complexity in relation to various predictor variables when accounting for phylogenetic interdependence using PGLS. All analyses were conducted on log10 transformed data.

| **Trait (α shape complexity)** | **λ** | **N** | **Predictor** | **Slope ± SE** | ***T*** | ***P*** |
| --- | --- | --- | --- | --- | --- | --- |
| All taxa | <0.01 | 18 | Neonate length | 0.51 ± 0.68 | 0.74 | 0.47 |
|  |  |  | Mother length | -0.33 ± 0.56 | -0.58 | 0.57 |
| All taxa | -0.22 | 18 | Testes mass | 0.04 ± 0.03 | 1.86 | 0.27 |
|  |  |  | Body mass | 0.00 ± 0.03 | 0.27 | 0.79 |
| All taxa | 0.26 | 18 | Sexual size dimorphism | -0.40 ± 0.28 | -1.43 | 0.17 |
| Cetaceans only | 0.7 | 12 | Neonate length | 1.15 ± 0.84 | 1.37 | 0.2 |
|  |  |  | Mother length | -1.05 ± 0.74 | -1.43 | 0.19 |
| Cetaceans only | 0.95 | 12 | Testes mass | -0.10 ± 0.04 | -2.74 | 0.02 |
|  |  |  | Body mass | 0.007 ± 0.04 | 0.18 | 0.86 |
| Cetaceans only | 0.44 | 12 | Sexual size dimorphism | -0.54 ± 0.38 | -1.43 | 0.18 |
