# Supplementary figures and images for: 3D genital shape complexity in female marine mammals
Source: Ecol Evol. 2021 Feb 15;11(7):3210–8. doi: 10.1002/ece3.7269 (PMC8019040; doi:10.1002/ece3.7269)

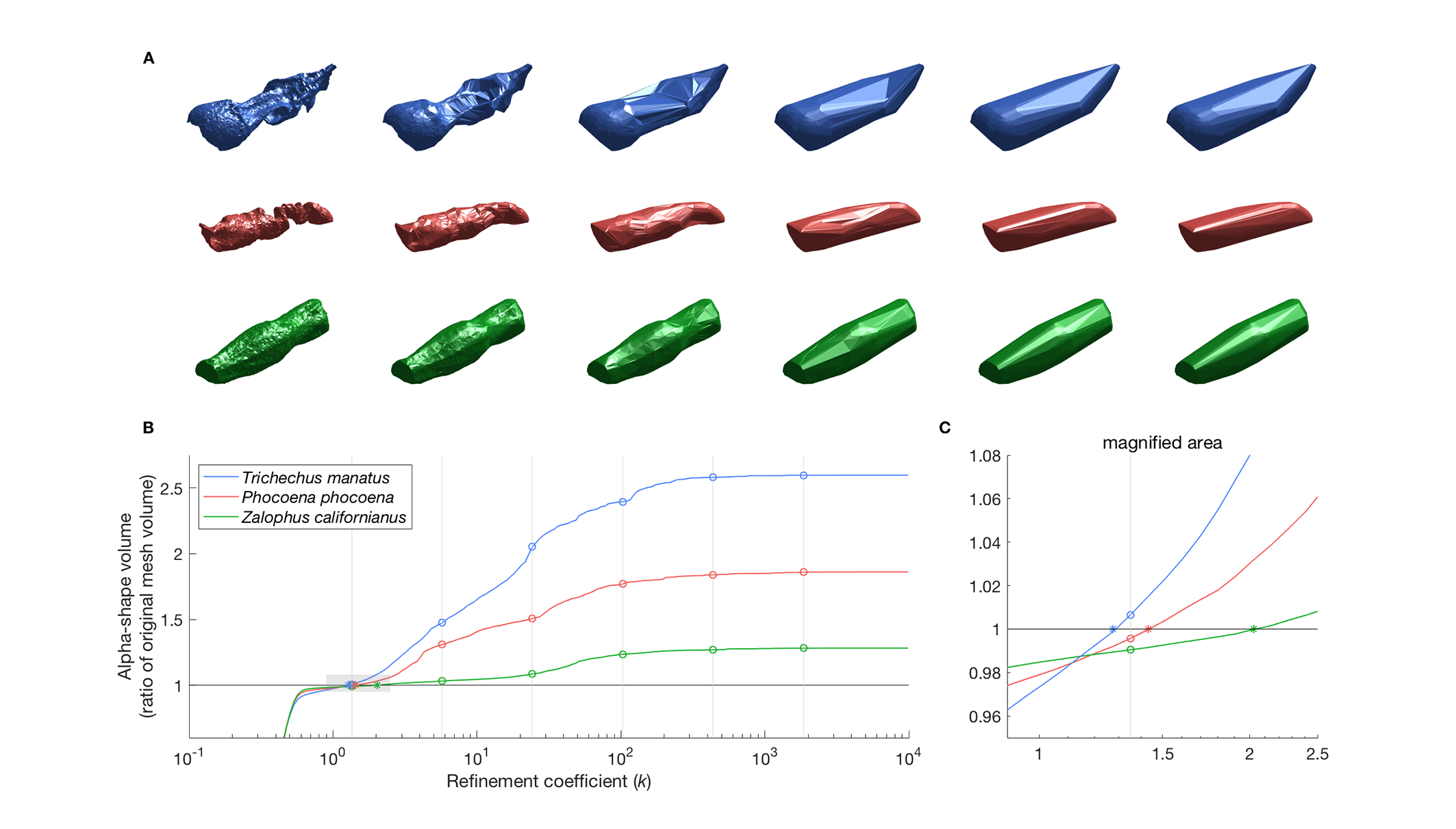

Supplement: Supplementary file 2 — Supplementary Material [file ECE3-11-3210-s002.tif]

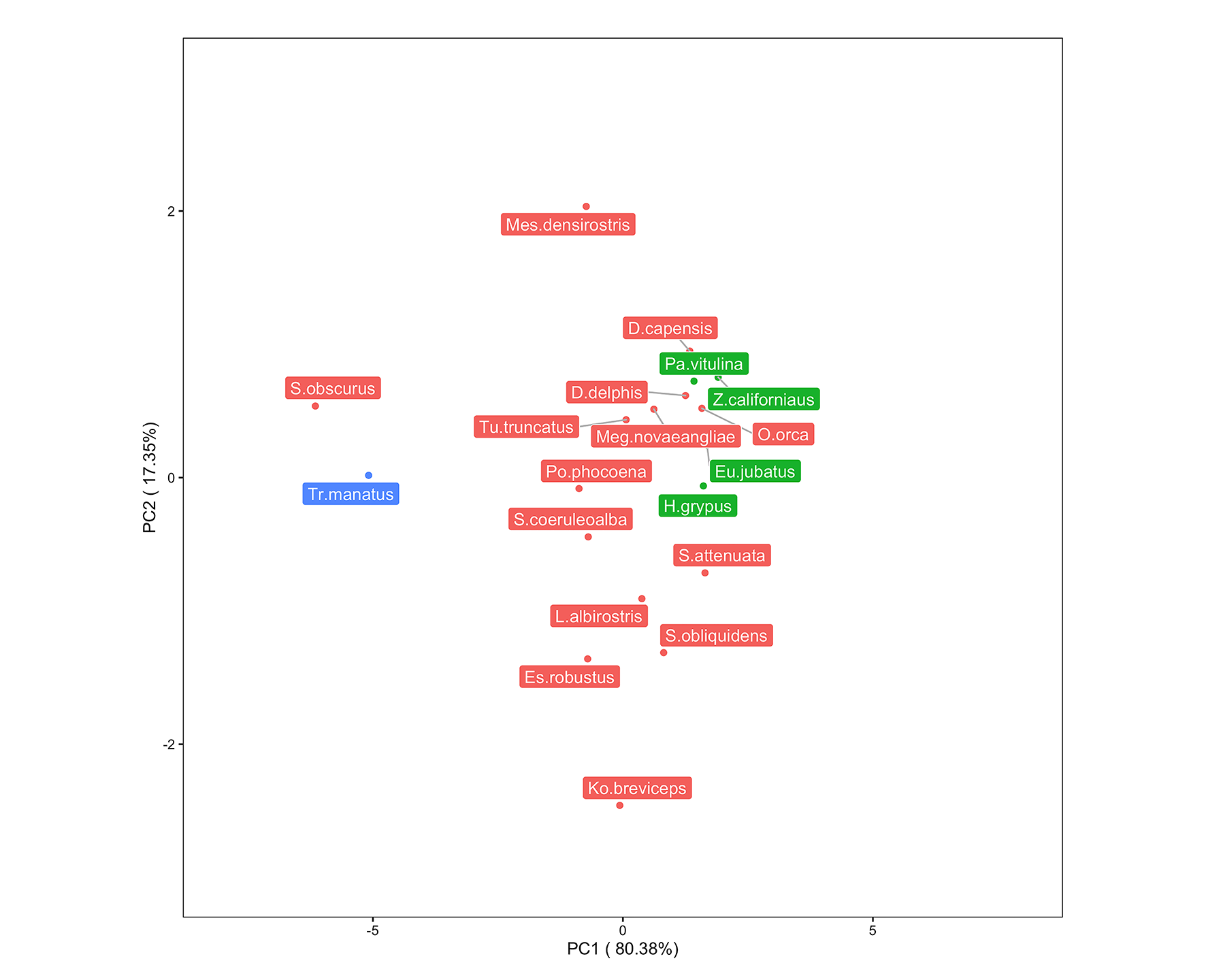

Supplement: Supplementary file 3 — Supplementary Material [file ECE3-11-3210-s001.tif]

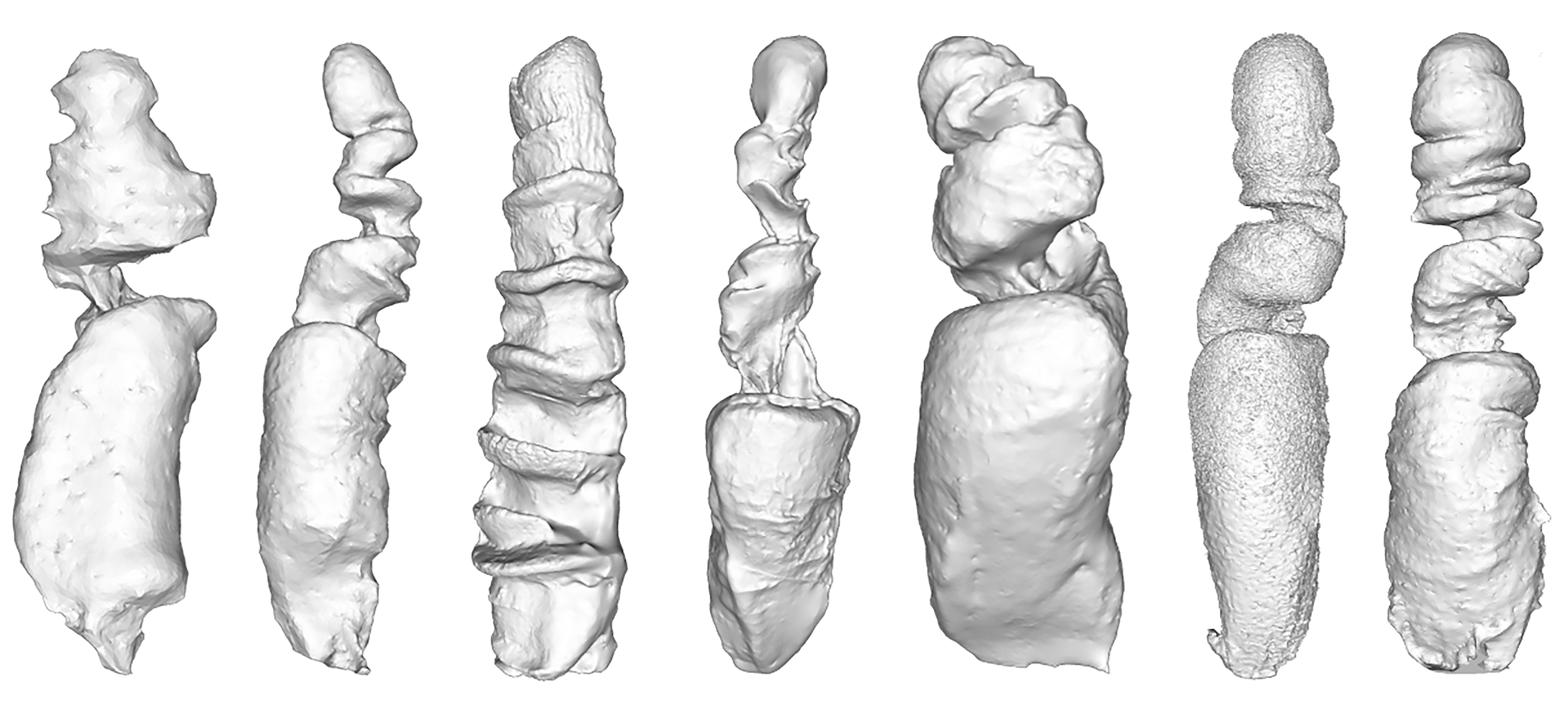

Supplement: Supplementary file 4 — Supplementary Material [file ECE3-11-3210-s003.tif]
